# MODIFIABLE DETERMINANTS OF PHYSICAL FUNCTIONING AMONG LGBT OLDER ADULTS WITH COGNITIVE IMPAIRMENT

**DOI:** 10.1093/geroni/igz038.1247

**Published:** 2019-11-08

**Authors:** Hyun-Jun Kim, Hyun-Jun Kim, Karen Fredriksen-Goldsen, and Hyunzee Jung

**Affiliations:** University of Washington, Seattle, Washington, United States

## Abstract

Heightened risks of cognitive impairment are a critical health concern for lesbian, gay, bisexual, and transgender (LGBT) older adults. The physical and functional declines associated with cognitive impairment are known to increase risks of injury and decrease independent mobility. Guided by the Health Equity Promotion Model, this study analyzed longitudinal data (T0 to T2, N=646) to examine risk and protective factors predicting changes in physical functioning over time among LGBT older adults with cognitive impairment. According to the results of multilevel mixed models, social support, physical, recreational, and wellness activities, and nutrition were associated with improvement in physical functioning over two years; while stigma contributed to decline in physical functioning. LGBT older adults with cognitive impairment face unique challenges due to social stigma and isolation. The findings suggest that enhancing their access to community resources and their participations in physical, social, community, and recreational activities would improve their physical functioning.

